# Association of Pericyte Loss With Microthrombosis After Subarachnoid Hemorrhage in ApoE-Deficient Mice

**DOI:** 10.3389/fneur.2021.726520

**Published:** 2021-09-10

**Authors:** Jinwei Pang, Yue Wu, Jianhua Peng, Ping Yang, Ligang Chen, Yong Jiang

**Affiliations:** ^1^Department of Neurosurgery, The Affiliated Hospital of Southwest Medical University, Luzhou, China; ^2^Department of Neurosurgery, The First Affiliated Hospital of Chongqing Medical University, Chongqing, China; ^3^Department of Cardiology, The Affiliated Hospital of Southwest Medical University, Luzhou, China; ^4^Sichuan Clinical Research Center for Neurosurgery, Luzhou, China; ^5^Luzhou Key Laboratory of Neurological Diseases and Brain Function, Luzhou, China

**Keywords:** subarachnoid hemorrhage, early brain injury, apolipoprotein E, pericytes, microthrombosis

## Abstract

**Background:** The occurrence of microthrombosis contributes to not only delayed cerebral ischemia (DCI), but also early brain injury (EBI) after SAH. However, the underlying mechanism is not completely investigated. In the current study, we explored the underlying mechanism of microthrombosis in EBI stage after SAH in ApoE-deficient mice.

**Methods:** Experimental SAH was established by endovascular perforation in apolipoprotein E (ApoE)-deficient mice and wild type (WT) mice. Neurobehavioral, molecular biological and histopathological methods were used to assess the relationship between pericytes loss, neurobehavioral performance, and microthrombosis.

**Results:** We found that the number of microthrombi was significantly increased and peaked 48 h after SAH in WT mice. The increased microthrombosis was related to the decreased effective microcirculation perfusion area and EBI severity. ApoE-deficient mice showed more extensive microthrombosis than that of WT mice 48 h after SAH, which was thereby associated with greater neurobehavioral deficits. Immunohistochemical staining showed that microthrombi were predominantly located in microvessels where pericytes coverage was absent. Mechanistically, ApoE deficiency caused more extensive CypA-NF-κB-MMP-9 pathway activation than that observed in WT mice, which thereby led to more degradation of N-cadherin, and subsequently more pericytes loss. Thereafter, the major adhesion molecule that promoting microthrombi formation in microvessels, P-selectin, was considerably increased in WT mice and increased to a greater extent in the ApoE-deficient mice.

**Conclusion:** Taken together, these data suggest that pericytes loss is associated with EBI after SAH through promoting microthrombosis. Therapies that target ApoE to reduce microthrombosis may be a promising strategy for SAH treatment.

## Introduction

Stroke is currently the second leading cause of death and disability worldwide (1). Despite accumulating knowledge of the disease pathology, treatments for stroke are limited (2). Although subarachnoid hemorrhage (SAH) represents approximately 5% of all strokes (3), it often affects people in a relatively young age with the mean age of time at SAH is around 50 years. It has a 35% mortality, and leaves many with lasting disabilities (4). The neurological outcomes of SAH patients are generally associated with the severity of initial bleeding, secondary brain injury, and/or medical complications (5). Early brain injury (EBI), which develops within the first 72 h after bleeding, in addition to cerebral vasospasm (CVS), is now believed to be the major predictor of early neurological deficits and long-term outcomes in SAH patients (6). However, the underlying mechanisms that contribute to EBI have not been fully identified. Therefore, therapeutics that completely cure EBI after SAH are still limited.

Microcirculatory dysfunction may contribute to delayed cerebral ischemia after SAH, but its role was largely ignored in the EBI stage after SAH. Microthrombosis was reported to induce cerebral ischemia, neurotoxic metabolic waste accumulation, cerebral edema, and neuronal apoptosis (7), which are all essential promoters of EBI and poor neurological outcomes after SAH. Clinically, the autopsies of SAH patients who died within 2 days after the initial bleeding event also showed a large number of microthrombi (8). Therefore, strategies that reduce microthrombosis in EBI after SAH may be beneficial for SAH treatment. Fortunately, the early administration of antiplatelet medications as well as anticoagulants has been suggested to be beneficial in improving neurological outcomes in some SAH patients (9, 10).

However, the mechanism by which microthrombi are induced after SAH remains incompletely understood. Previously, apolipoprotein E (ApoE: protein, APOE: gene) was shown to affect platelet activation (11), which is an important part of microthrombosis. Our recent study found that ApoE deficiency is associated with more significant EBI and neurological impairments after SAH (12). However, the underlying mechanisms have not been fully identified. In clinical studies, the lack of an association between the APOE epsilon4 allele and signs of visible angiography vasospasm but the presence of an association between the allele and an increased risk of delayed cerebral ischemia after SAH have linked ApoE to the cerebral microcirculation, probably through coagulation and fibrinolytic cascade impairment and microthrombosis (13).

In the current study, we evaluated behavioral, histological and molecular biological data to compare microthrombosis in WT and ApoE-deficient mice following SAH and explored the underlying mechanisms.

## Materials and Methods

### Animals

All animal experiments were approved by the Ethics Committee of Southwest Medical University and carried out in accordance with Stroke Treatment and Academic Roundtable (STAIR) guidelines and the National Institutes of Health Guide for the Care and Use of Laboratory Animals. Healthy young adult male wild-type C57BL/6J mice (WT; 8–10 weeks; 20–25 g) and physiological condition-matched ApoE-deficient mice on a C57BL/6J background (KO; 8–10 weeks; 20–25 g) were used. The animals were housed and humanely cared for in the Laboratory Animal Resource Center (LARC) and allowed free access to food and water.

### SAH Model Establishment

Experimental SAH was induced using the endovascular perforation technique as described previously (14). Briefly, the mice were anesthetized with 2% pentobarbital (50 mg/kg) by intraperitoneal injection. A 5-0 monofilament was inserted via the right external carotid artery to perforate the bifurcation of the middle and anterior cerebral artery. SAH was confirmed by an obvious Cushing response and autopsy after sacrifice at scheduled time points. In the sham-operated animals, all surgical procedures were repeated except SAH induction by vessel puncture. The mice were warmed by an electric blanket, and the rectal temperature was maintained at 37.5 ± 0.5°C during the surgery. The mice were administered 50 mg/kg ampicillin in 0.9% saline twice daily after surgery until sacrifice. The mice that died due to severe SAH were immediately replaced with condition-matched animals.

### Brain Water Content Calculation

Brain water content was measured after neurological function evaluation as previously reported (15). The animals were euthanized with an overdose of pentobarbital sodium. The brains were harvested, and the left and right hemispheres were dissected for brain water content measurement. The wet weight was immediately recorded, and the dry weight was acquired after drying at 100°C for 72 h. The brain water content was calculated as (wet weight–dry weight)/wet weight × 100%.

### Rota Rod Latency

To evaluate mouse neurological performance, Rota Rod latency was assessed in all animals (*n* = 15–22 for each experiment) using an automated Rota Rod device (ZB-200 Rota Rod Treadmill; Taimeng Software Co. LTD, Chengdu, China) by a blinded investigator, as previously reported (16). All animals completed a total of 9 training sessions over a period of 3 days before surgery. Baseline Rota Rod latency were assessed three times for each group before SAH induction using the accelerating mode (the rotating speed started at 0 rpm and was accelerated by 3 rpm every 10 s until it reached 30 rpm), and Rota Rod performance was assessed again after SAH. The Rota Rod latency was defined as the average latency of all three trials.

### Effective Microcirculation Perfusion Area Analysis

Intravital lectin perfusion was used to identify the effective microcirculation perfusion area without microthrombi after SAH. Briefly, 1 mg/ml biotin-labeled Lycopersicon esculentum (Tomato) lectin (LEL) was dissolved in PBS, and the mice received a 400 μl intravenous lectin injection 1 h before sacrifice. Brain samples were collected routinely, and 10-μm coronal frozen sections were made. The sections were rinsed three times in PBS and then incubated with AMCA-streptavidin (SA-5008, Vector Laboratories) for 1 h at room temperature. After rising and mounting, the sections were visualized under a fluorescence microscope (Olympus, Tokyo, Japan). Three nonadjacent coronary sections from each brain sample with a minimum distance of 100 μm from one another were used. Five randomly selected visual fields per section were observed and analyzed by a blinded observer using Image-Pro Plus (IPP) 6.0 software.

### Western Blot Analyses

Protein from each right hemisphere sample was extracted, quantified, and denatured at 95°C in 5X loading buffer for 10 min. Western blot was performed as previously described (17). The following primary antibodies were used: Fibrin (ogen) (ab34269, Abcam), P-selectin (60322-1-Ig, Proteintech), PDGFRβ (ab69506, Abcam), CypA (ab41684, Abcam), N-cadherin (22018-1-AP, Proteintech), phospho-NF-κB p65 subunit (p-p65, ab86299, Abcam), MMP-9 (10375-2-AP, Proteintech), and β-actin (66009-1-Ig, Proteintech). The bands were visualized using a BeyoECL Plus kit (P0018; Beyotime) and photographed by a chemiluminescence imaging system (ChemiDoc XRS+; Bio-Rad, Hercules, CA, USA). Band densities were quantified by a blinded observer using ImageJ software.

### Microthrombi Staining

Microthrombi were detected by immunohistochemical staining for fibrin (ogen) according to a previous study (18). Brain samples were routinely collected, and 10-μm coronal frozen sections were made. The sections were rinsed three times in PBS and then incubated with 3% hydrogen peroxide for 10 min at room temperature. Thereafter, the sections were washed again and blocked with 5% normal goat serum for 30 min at room temperature and then incubated with a fibrin (ogen) primary antibody (ab34269, Abcam) at 4°C overnight. After washing with PBS, the sections were incubated with a biotinylated goat anti-rabbit secondary antibody for 1 h at room temperature and then analyzed by an HRP/DAB IHC detection system. Three nonadjacent coronary sections from each brain sample with a minimum distance of 100 μm from one another were used. Five randomly selected visual fields per section were observed and analyzed using Image-pro plus (IPP) 6.0 software by a blinded observer.

### Immunofluorescent Staining

For immunofluorescent staining, brain samples were routinely collected, and 10-μm coronal frozen sections were made. The immunofluorescent staining procedure was performed as previously reported (19). The following primary antibodies were used: PDGFR-β (ab32570, Abcam), biotinylated lectin (B-1175, Vector Laboratories), fibrin (ogen) (ab34269, Abcam) and P-selectin (60322-1-Ig, Proteintech). Secondary antibodies, including AMCA-streptavidin (SA-5008, Vector Laboratories), DyLight 488 goat anti-rabbit IgG (A23220, Abbkine), and DyLight 594 goat anti-mouse IgG (A23410, Abbkine) were used. Three nonadjacent coronary sections from each brain sample with a minimum distance of 100 μm from one another were used. Five randomly selected visual fields per section were observed and analyzed by a blinded observer using Image-pro plus (IPP) 6.0 software by a blinded observer.

### Statistical Analysis

The normally distributed quantitative data are expressed as the mean ± SD. For normally distributed data, one-way analysis of variance (ANOVA) with Tukey's *post-hoc* test was used to compare the means of the different groups. A *p* < 0.05 was considered statistically significant. Mortality analysis was performed by Fisher's exact test. Correlation analysis between two parameters was tested by the Pearson correlation test. All statistical values were analyzed using SPSS 20.0 software (SPSS, Inc. Chicago, IL, USA).

## Results

### SAH Severity and Animal Mortality

The SAH grade of the WT mice from each time course subgroup and the ApoE-deficient mice were not significantly different. No animal from the WT-Sham or KO-Sham groups died. The overall mortality rate was 14.7% (13 of 88) in the WT group and 21.05% (8 of 38) in the KO group. Although the mortality in the KO group was higher than that in the WT group, there was no significant difference in mortality between these groups.

### The Number of Microthrombi Was Increased in EBI Stage After SAH

**Figure 1:**
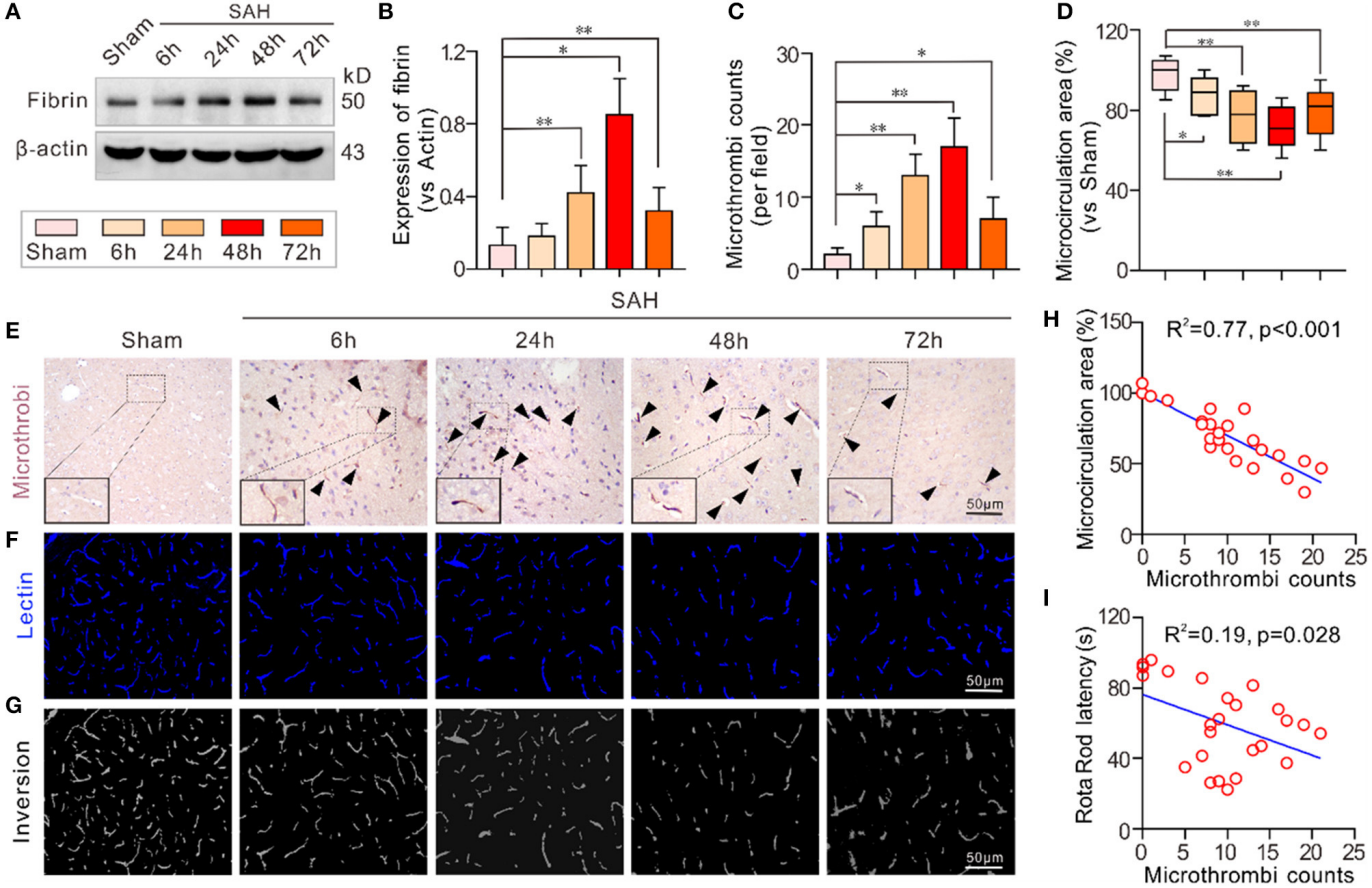
Microthrombosis was increased in EBI after SAH. **(A,B)** The expression of the microthrombi marker fibrinogen gradually increased at different time points and peaked 48 h after SAH. **(C,D)** The quantification of the number of microthrombi and effective microcirculation perfusion area. **(E)** Representative pictures of the fibrinogen-positive microthrombi at each time point. **(F)** Representative pictures of the intravital lectin perfusion study for evaluating the effective microcirculation area. **(G)** Grayscale inversion images corresponding to the pictures shown in **(E)**. **(H,I)** The Pearson correlation test between the number of microthrombi, and effective microcirculation area and neurobehavioral performance. Except for Pearson correlation analysis, the data are expressed as the mean ± SD, one-way ANOVA with Tukey's *post hoc*, *p < 0.05, ^**^p < 0.01, vs. Sham group; *n* = 5 per group; Fibrin, fibrinogen; scale bar = 50 mm.

The expression of the microthrombi marker fibrinogen was significantly increased and peaked 48 h after SAH compared to the sham group. Even at 72 h, the expression of fibrinogen was still higher than that in the sham group (Figures 1A,B). Immunochemical staining of fibrinogen also showed a similar trend in the number of microthrombi (Figures 1C,E). Conversely, intravital lectin perfusion showed that the effective microcirculatory perfusion area gradually decreased until 48 h after SAH and then increased toward baseline levels 72 h after SAH (Figures 1D,F,G). The Pearson correlation test showed that the number of microthrombi was closely correlated with neurological performance in the mice (Rota Rod latency) (*R*^2^ = 0.19, *p* = 0.028) and the effective microcirculatory area (*R*^2^ = 0.77, *p* < 0.001; Figures 1H,I).

### Effective Microcirculation Area Was Reduced in ApoE Deficient Mice After SAH

**Figure 2:**
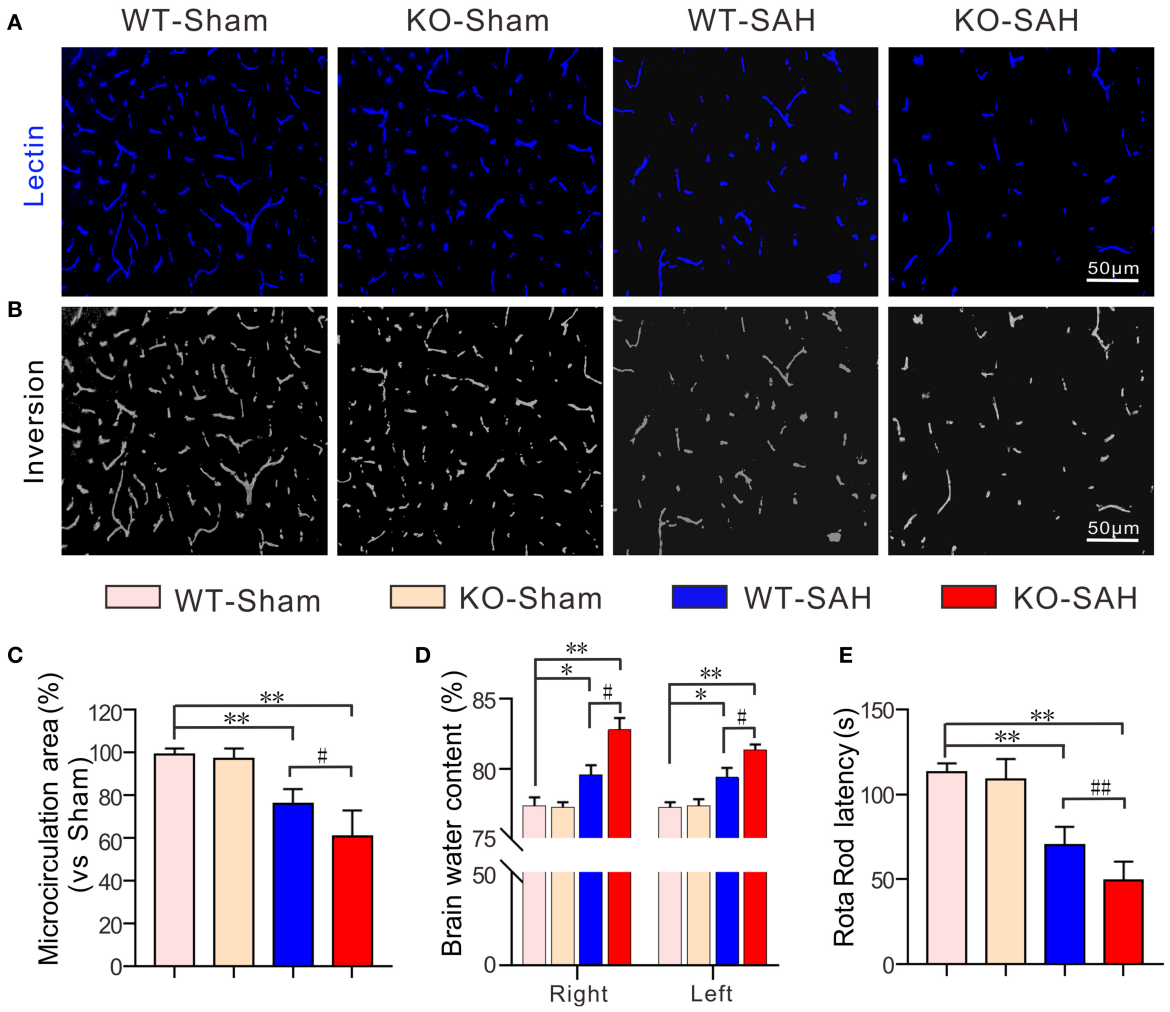
ApoE deficiency promotes effective microcirculation perfusion area reduction after SAH. **(A)** Representative pictures of the intravital lectin perfusion study for evaluating the effective microcirculation area in WT mice and ApoE-deficient mice 48 h after SAH. **(B)** Corresponding grayscale inversion images of the pictures shown in **(A)**. **(C)** The quantification of the effective microcirculation perfusion area in each group. **(D)** The quantification of brain water content in each group. **(E)** The quantification of the rotarod latency in each group. Data are expressed as the mean ± SD, one-way ANOVA with Tukey's *post hoc*, *p < 0.05, **p < 0.01, vs. the WT-Sham group; ^#^*p* < 0.05, vs. the WT-SAH group; *n* = 5 per group; Right, right hemisphere; Left, left hemisphere; scale bar = 50 mm.

In both the WT-Sham and KO-Sham mice, the effective microcirculation perfusion areas observed in the intravital lectin perfusion study were not significantly different. However, 48 h after SAH, the effective microcirculation perfusion area was significantly reduced in both the WT-SAH group and KO-SAH group. The reduction in the effective microcirculation perfusion area was dramatically higher in the ApoE-deficient mice 48 h after SAH (Figures 2A–C). The greater reduction in microcirculation perfusion was associated with more severe brain edema and lower Rota rod latency in the ApoE-deficient mice than in the WT mice (Figures 2D,E).

### Pericytes Loss Associated With Microthrombosis in ApoE-Deficient Mice After SAH

**Figure 3:**
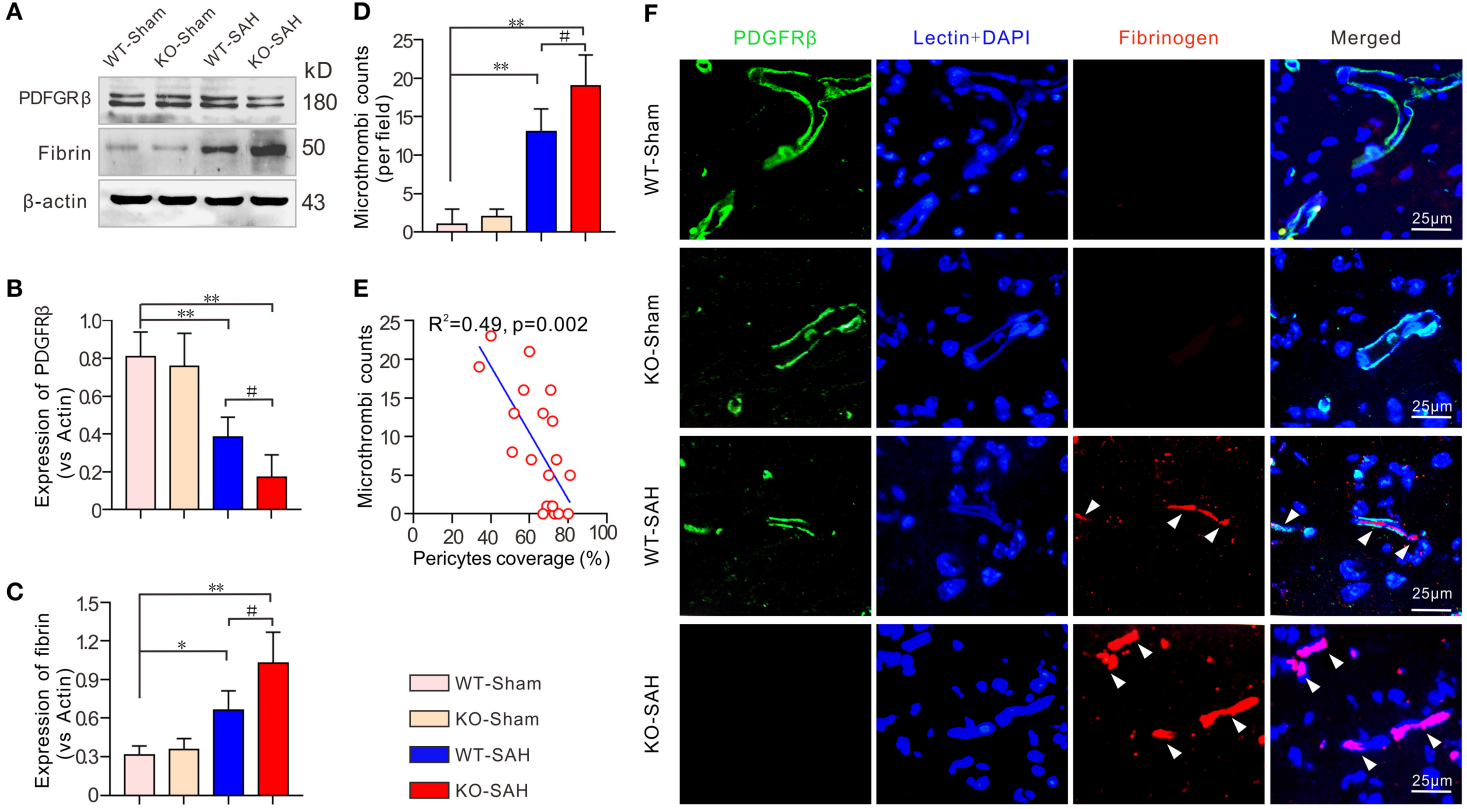
Pericytes loss promoted icrothrombosis in ApoE-deficient mice after SAH. **(A)** Immunoblotting analysis of the expression of the pericytes marker PDGFRβ and the microthrombi marker fibrinogen in each group. **(B,C)** The quantification of PDGFRβ and fibrinogen. **(D)** Quantification of the microthrombi in each group 48 h after SAH. **(E)** Pearson correlation test between pericytes coverage and microthrombi number in each group. **(F)** Immunofluorescent triple staining with lectin + PDGFRβ+ fibrinogen showed that microthrombi were primarily located where PDGFRβ-positive pericytes were missing. Except for Pearson correlation analysis, data are expressed as the mean ± SD, one-way ANOVA with Tukey's *post hoc*, *p < 0.05, **p < 0.01, vs. the WT-Sham group; ^#^*p* < 0.05, vs. the WT-SAH group; *n* = 5 per group; Fibrin, fibrinogen; scale bar = 25 mm.

Prior to SAH, no significant difference was observed in the expression of PDGFRβ and fibrinogen between the WT-Sham group and the KO-Sham group. However, 48 h after SAH, the mice in the WT-SAH group showed a significant decrease in PDGFRβ expression, which was accompanied by a significant increase in fibrinogen expression. These changes were increased to an even greater extent in the ApoE-deficient mice (Figures 3A–C). Immunofluorescent staining showed that no obvious microthrombi were observed in either the WT-Sham group or the KO-Sham group, but abundant microthrombi was observed in WT-SAH, and the number of microthrombi was increased to an even greater extent in the ApoE-deficient mice. Notably, the microthrombi were mainly located in sites where PDGFRβ-positive pericytes were absent, especially in the ApoE-deficient mice (Figures 3D,F). The Pearson correlation test showed that the number of microthrombi was closely correlated with pericytes coverage (*R*^2^ = 0.49, *p* = 0.002; Figure 3E).

### N-Cadherin Degradation Promoted Pericytes Loss and P-Selectin Elevation in EBI Stage After SAH

**Figure 4:**
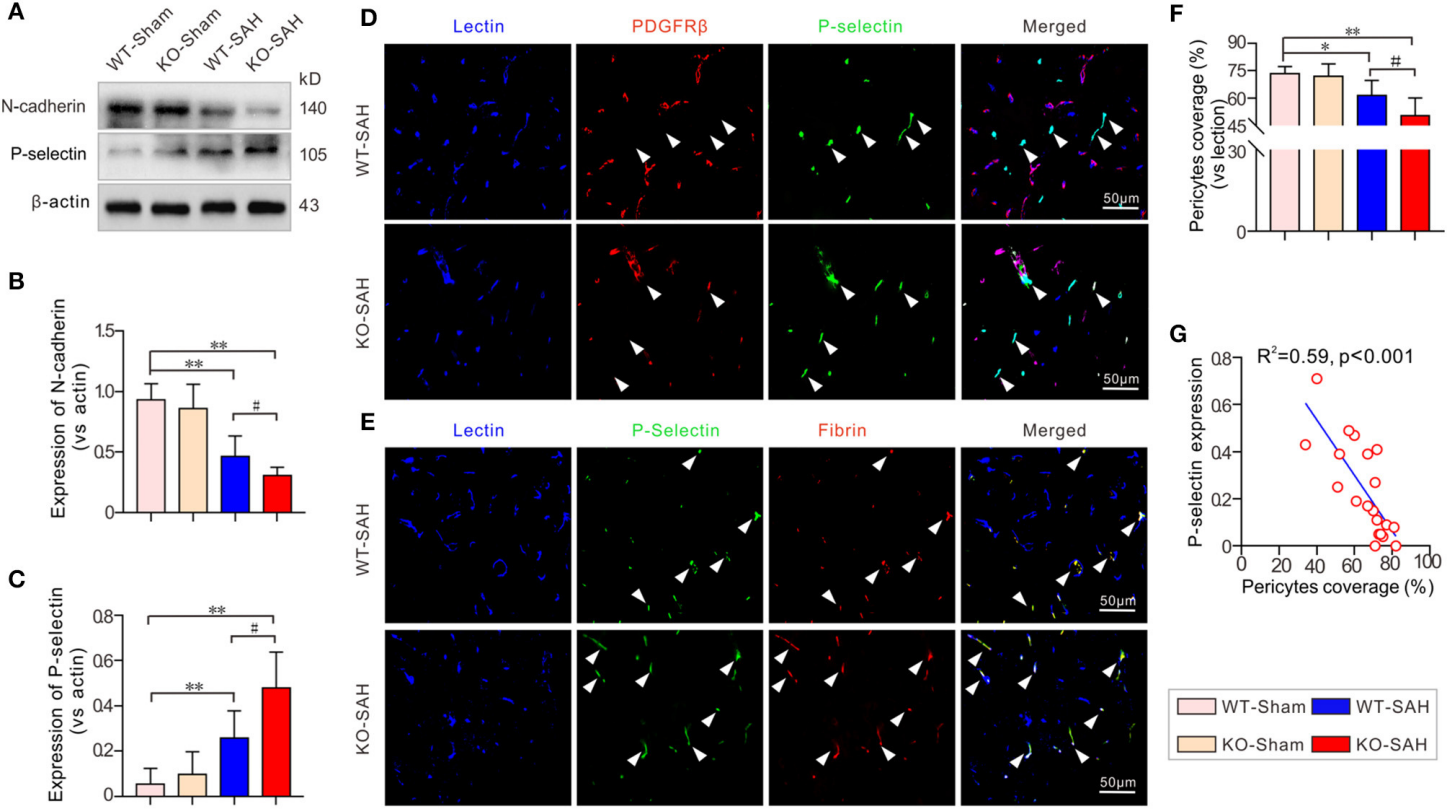
N-cadherin degradation promoted pericyte loss and P-selectin elevation in EBI stage after SAH. **(A)** Immunoblotting analysis of the expression of N-cadherin and P-selectin. **(B,C)** The quantification of N-cadherin and P-selectin. **(D)** Representative pictures of immunofluorescent triple-staining with lectin + PDGFRβ + P-selectin. **(E)** Representative pictures of immunofluorescent triple-staining with lectin + P-selectin + fibrinogen. The results showed that P-selectin was primarily localized to fibrinogen-positive microthrombi and was mainly located in locations where PDGFRβ-positive pericytes were absent. **(F)** The quantification of the pericytes coverage change in each group 48 h after SAH. **(G)** The Pearson correlation test between pericytes coverage and P-selectin expression. Except for Pearson correlation analysis, the data are expressed as the mean ± SD, one-way ANOVA with Tukey's *post hoc*, *p < 0.05, **p < 0.01, vs. the WT-Sham group; ^#^*p* < 0.05, vs. the WT-SAH group; *n* = 5 per group; Fibrin, fibrinogen; scale bar = 50 mm.

Prior to SAH, no significant difference was observed in the expression of N-cadherin, the major tight junction molecule between endothelial cells and pericytes, between the WT-Sham group and the KO-Sham group. Additionally, the expression of the major promoter of microthrombosis, P-selectin, was also not obviously different between the WT-Sham group and the KO-Sham group. However, 48 h after SAH, N-cadherin expression was significantly decreased in the WT mice, which resulted in a significant loss of pericytes coverage and an increase in P-selectin expression. These changes were more severe in the ApoE-deficient mice (Figures 4A–C). Further analysis by immunofluorescent staining showed that pericytes coverage was significantly reduced after SAH, and P-selectin was mainly expressed by endothelial cells in areas where pericytes were absent, and co-localized with microthrombi marker fibrinogen (Figures 4D–F). The Pearson correlation test showed that P-selectin expression was closely correlated with pericytes coverage (*R*^2^ = 0.59, *p* < 0.001; Figure 4G).

### The CypA-NF-κB-MMP-9 Pathway Was Activated in EBI Stage After SAH

**Figure 5:**
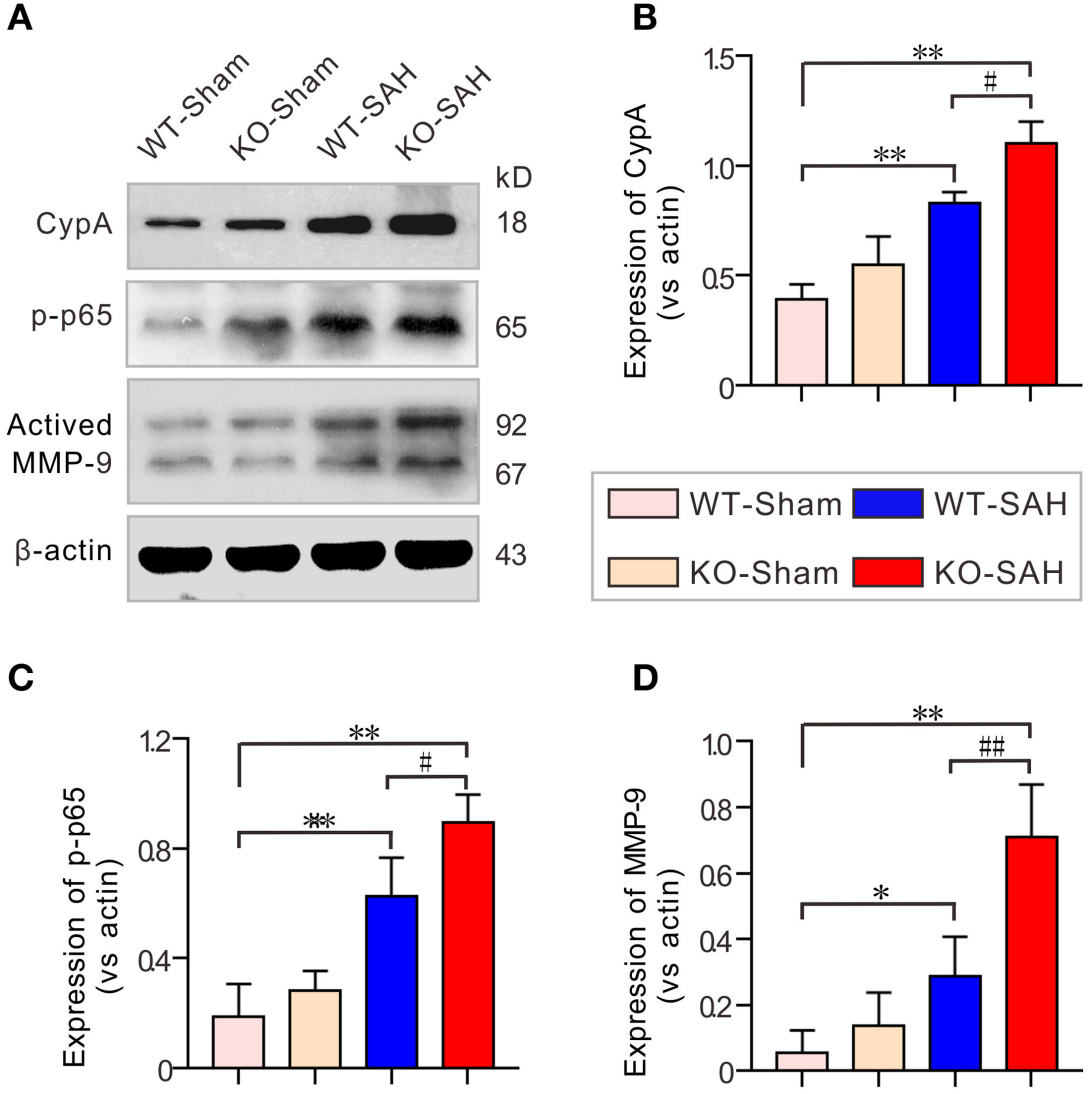
The CypA-NF-κB-MMP-9 pathway was activated in EBI after SAH. **(A)** Immunoblotting analysis of CypA, p-p65 and MMP-9 expression in each group 48 h after SAH. **(B–D)** The quantification of CypA, p-p65, and MMP-9. The data are expressed as the mean ± SD, one-way ANOVA with Tukey's *post hoc*, *p < 0.05, **p < 0.01, vs. the WT-Sham group; ^#^*p* < 0.05, ^*##*^*p* < 0.01, vs. the WT-SAH group; *n* = 5 per group.

A previous study showed that the CypA-NF-κB-MMP-9 pathway can regulate the integrity of the BBB via affecting the interactions between endothelial cells and pericytes (20). In line with our previous work (12), our results showed that the expression of CypA and phosphorylated NF-κB p65 subunit (p-p65) was slightly higher in the sham-operated ApoE-deficient mice, but there was no significant difference when this group was compared to the WT-Sham mice. However, CypA, p-p65, and MMP-9 were dramatically upregulated 48 h after SAH, with larger increases of these proteins in the ApoE-deficient mice (Figure 5).

## Discussion

The microcirculation plays a pivotal role in the maintenance of cerebral homeostasis and neuronal metabolite exchange (21, 22). However, after SAH, the microcirculation can be destroyed due to increased BBB disruption and microthrombosis, which result in cerebral ischemia, neuronal nutrient deprivation and severe brain edema, which subsequently induces neuronal death (23–25). Hence, cerebral microcirculation dysfunction has been demonstrated to be an important part of secondary pathogenic changes after SAH. However, despite microcirculatory thrombosis has been reported to affect secondary brain injury after SAH, little is known about how and why it forms. Therefore, a better understanding of the underlying mechanism involved in cerebral microcirculatory thrombosis is warranted for improving SAH treatment.

The current study explored microthrombosis and the associated underlying mechanism in the EBI stage after experimental SAH using ApoE-deficient mouse. First, to further identify the importance of microthrombosis in EBI, we analyzed the formation of microthrombi and the correlation of microthrombi with neurobehavioral performance and the effective microcirculatory perfusion area. The fact that microthrombi increase in our study were similar with the fact that microthrombi was also increased in brain autopsies of SAH patients (26). Our results showed that the number of microthrombi was significantly increased after SAH, while the intravital lectin perfusion study showed that the effective microcirculatory perfusion area was greatly decreased after SAH. The Pearson correlation test showed that number of microthrombi was closely correlated with neurological performance in the mice (Rota Rod latency) (*R*^2^ = 0.19, *p* = 0.028) and the effective microcirculatory area (*R*^2^ = 0.77, *p* < 0.001).

Furthermore, when compared with the WT mice, there was no microthrombi in the sham-operated ApoE-deficient mice, but the expression of the microthrombi marker fibrinogen and the number of microthrombi were more significantly increased in the ApoE-deficient mice at 48 h after SAH. Brain edema, the effective microcirculatory perfusion area and neurobehavioral performance were also changed more markedly in the ApoE-deficient mice at this time point. Thus, ApoE gene knockout is associated with increased microthrombosis in EBI after SAH. These findings are consistent with our previous data showing that EBI is more severe in ApoE-deficient mice after SAH (12).

Although the number of microthrombi was increased in EBI after SAH and was further increased in ApoE-deficient mice, the mechanisms by which microthrombosis is affected after SAH remain elusive. P-selectin belongs to the adhesion glycoprotein family, which mediates leukocyte-endothelial cell and leukocyte-platelet adhesive interactions (27). It can be significantly upregulated and translocated to the surface of platelets and endothelial cells to promote vascular changes in many microcirculatory disturbance events. It has been reported that the increased expression of P-selectin in endothelial cells, which facilitates platelet adhesion, is the main cause of microthrombosis after traumatic brain injury (28).

In SAH studies, the increased expression of P-selectin in endothelial cells, which subsequently facilitates platelet adhesion, has also been reported to be the main cause of microthrombosis (29). ApoE deficiency has been reported to increase P-selectin expression in various situations. Therefore, the ApoE deficiency-induced increase in microthrombosis after SAH may be related to an increase in P-selectin expression. We then checked the expression of P-selectin after SAH in WT mice and ApoE-deficient mice. In the current study, P-selectin expression was significantly upregulated after SAH in WT mice and upregulated to a greater extent in ApoE-deficient mice. Moreover, as observed by immunofluorescent staining, P-selectin was mainly expressed by endothelial cells in the presence of abundant microthrombi. Thus, understanding how P-selectin expression is regulated may contribute to understanding the mechanism of microthrombosis after SAH.

Pericytes, as an important cellular component of the microvasculature, are necessary for neuronal homeostasis and the maintenance of the cerebral microcirculation. Traditionally, pericyte, served as capillary contraction handler, is recently considered as the main participant of microcirculation regulation in SAH pathophysiology (30). Although cerebral pericytes loss was involved in the dysfunction of microcirculation in EBI after SAH (31), this role has been largely overlooked.

In the current study, most of the microvessels were covered with pericytes in both the WT-Sham mice and KO-Sham mice. However, 48 h after SAH, the pericytes coverage was significantly reduced in the WT-SAH group and further decreased in the ApoE-deficient mice. Immunofluorescent staining showed that P-selectin was primarily colocalized with fibrinogen-positive microthrombi and was mainly located in areas where PDGFRβ-positive pericytes were absent, both in the WT mice and the ApoE-deficient mice. In addition, P-selectin and microthrombi were more frequently observed due to lower microvessel pericytes coverage 48 h after SAH in the ApoE-deficient mice. These data suggest that SAH-induced pericytes loss can lead to the upregulation of P-selectin expression and thereby facilitate microthrombosis. ApoE deficiency can further promote pericytes loss and subsequently induce more microthrombosis in EBI after SAH Thus, knowing how pericytes loss occurs after SAH and ApoE deficiency can help determine the mechanism of microthrombosis after SAH.

Given that pericytes-endothelial cell interactions are mainly controlled by the N-cadherin complex (32), we then measured the expression of N-cadherin and its upstream regulators to further identify the mechanism that promotes pericytes loss and microthrombosis after SAH. The results showed that the N-cadherin protein level was significantly reduced after SAH and decreased to a greater extent in the ApoE-deficient mice. It has been reported that MMP-9 activation can lead to N-cadherin degradation and thereby promote pericytes and endothelial cell decoupling (33, 34). Our previous study reported that CypA-NF-κB pathway-induced MMP-9 activation is associated with BBB tight junction protein degradation and EBI formation (35). We then measured the protein levels of CypA, NF-κB, and MMP-9. The results showed that both CypA, phosphorylated NF-κB subunit p65 (p-p65) and MMP-9 were dramatically upregulated 48 h after SAH, with greater increases in these proteins being observed in ApoE-deficient mice, whereas the expression of N-cadherin showed the opposite trend. Therefore, N-cadherin protein degradation is likely mediated by the activation of the CypA-NF-κB- MMP-9 pathway.

Taken together, the present findings support the hypothesis that pericytes loss is associated with EBI after SAH through promoting microthrombosis. ApoE deficiency can lead to a greater loss of pericytes and subsequently lead to more microthrombi and a more severe EBI. This phenomenon is mediated at least partly by the activation of CypA-NF-κB-MMP-9 signaling pathway-dependent N-cadherin degradation. Therapies that targeting ApoE to reduce microthrombosis may be a promising strategy for SAH treatment.

Although our findings are informative for future studies of SAH, several limitations of this study should be noted. First, it was reported that women have a 1.6 times higher risk of SAH than men (36), and estrogen has an impact on coagulation and fibrinolytic systems (37). The current study used only young male mice. Whether gender difference affect the effects of ApoE on pericytes loss and microthrombosis after SAH is not clear. In addition, although we found that pericytes loss is associated with an increase in P-selectin and microthrombosis in ApoE-deficient mice after SAH, the direct mechanism that upregulates P-selectin after SAH is still not clear. Furthermore, this study focused on the effect of ApoE on microthrombosis in the EBI stage after SAH, but no long-term outcomes were evaluated. Whether ApoE deficiency-related microthrombosis is associated with long-term outcomes remains unclear. Therefore, further investigation is still needed to completely clarify the effect of ApoE on SAH induced secondary brain injuries.

## Data Availability Statement

The original contributions presented in the study are included in the article/supplementary material, further inquiries can be directed to the corresponding author/s.

## Ethics Statement

The animal study was reviewed and approved by Ethics Committee of Southwest Medical University.

## Author Contributions

JPa and YW conceived and designed the study, performed the experiments, and wrote the manuscript. JPe contributed to the acquisition and/or interpretation of data. JPe and PY contributed to manuscript writing. LC and YJ made substantial contributions to fund the study. All authors contributed to the article and approved the submitted version.

## Funding

This work was supported by the National Natural Science Foundation of China (81801176, 81771278, and 81971132) and the Sichuan Science and Technology Program (2019JDRC0062 and 2019JDTD0004).

## Conflict of Interest

The authors declare that the research was conducted in the absence of any commercial or financial relationships that could be construed as a potential conflict of interest. The reviewer ZX declared a shared affiliation, with no collaboration, with one of the authors YW to the handling Editor.

## Publisher's Note

All claims expressed in this article are solely those of the authors and do not necessarily represent those of their affiliated organizations, or those of the publisher, the editors and the reviewers. Any product that may be evaluated in this article, or claim that may be made by its manufacturer, is not guaranteed or endorsed by the publisher.
